# Shared Risk Factors for Depressive Disorder Among Older Adult Couples in Korea

**DOI:** 10.1001/jamanetworkopen.2023.8263

**Published:** 2023-04-14

**Authors:** Ji Won Han, Hee Won Yang, Jong Bin Bae, Dae Jong Oh, Dong Gyu Moon, Eunji Lim, Jin Shin, Bong Jo Kim, Dong Woo Lee, Jeong Lan Kim, Jin Hyeong Jhoo, Joon Hyuk Park, Jung Jae Lee, Kyung Phil Kwak, Seok Bum Lee, Seok Woo Moon, Seung-Ho Ryu, Shin Gyeom Kim, Ki Woong Kim

**Affiliations:** 1 Department of Neuropsychiatry, Seoul National University Bundang Hospital, Seongnam, South Korea; 2 Department of Psychiatry, Seoul National University, College of Medicine, Seoul, South Korea; 3 Department of Psychiatry, Chungnam National University Hospital, Daejeon, South Korea; 4 Workplace Mental Health Institute, Kangbuk Samsung Hospital, Sungkyunkwan University School of Medicine, Seoul, South Korea; 5 Department of Neuropsychiatry, Gyeongsang National University Changwon Hospital, Changwon, Republic of Korea; 6 Department of Psychiatry, Gyeongsang National University, School of Medicine, Jinju, South Korea; 7 Department of Neuropsychiatry, Inje University Sanggye Paik Hospital, Seoul, South Korea; 8 Department of Psychiatry, School of Medicine, Chungnam National University, Daejeon, South Korea; 9 Department of Neuropsychiatry, Kangwon National University Hospital, Chuncheon, South Korea; 10 Department of Neuropsychiatry, Jeju National University Hospital, Jeju, South Korea; 11 Department of Psychiatry, Dankook University Hospital, Cheonan, South Korea; 12 Department of Psychiatry, Dongguk University Gyeongju Hospital, Gyeongju, South Korea; 13 Department of Psychiatry, School of Medicine, Konkuk University and Konkuk University Chungju Hospital, Chungju, South Korea; 14 Department of Psychiatry, School of Medicine, Konkuk University and Konkuk University Medical Center, Seoul, South Korea; 15 Department of Neuropsychiatry, Soonchunhyang University Bucheon Hospital, Bucheon, South Korea; 16 Department of Brain and Cognitive Science, Seoul National University College of Natural Sciences, Seoul, South Korea

## Abstract

**Importance:**

Although couples may share many risk factors for depressive disorders in their lifetime, whether these factors mediate the shared risk of depressive disorders has rarely been investigated.

**Objectives:**

To identify the shared risk factors for depressive disorder in couples and investigate their mediating roles in the shared risk of depressive disorders among older adult couples.

**Design, Setting, and Participants:**

This nationwide, multicenter, community-based cohort study assessed 956 older adults from the Korean Longitudinal Study on Cognitive Aging and Dementia (KLOSCAD) and a cohort of their spouses (KLOSCAD-S) between January 1, 2019, to February 28, 2021.

**Exposures:**

Depressive disorders of the KLOSCAD participants.

**Main Outcomes and Measures:**

The mediating roles of shared factors in couples on the association between one spouse’s depressive disorder and the other’s risk of depressive disorders was examined using structural equation modeling.

**Results:**

A total of 956 KLOSCAD participants (385 women [40.3%] and 571 men [59.7%]; mean [SD] age, 75.1 [5.0] years) and their spouses (571 women [59.7%] and 385 men [40.3%]; mean [SD] age, 73.9 [6.1] years) were included. The depressive disorders of the KLOSCAD participants were associated with an almost 4-fold higher risk of depressive disorders in their spouses in the KLOSCAD-S cohort (odds ratio, 3.89; 95% CI, 2.06-7.19; *P* < .001). Social-emotional support mediated the association between depressive disorders in the KLOSCAD participants and their spouses’ risk of depressive disorders by itself (β = 0.012; 95% CI, 0.001-0.024; *P* = .04; mediation proportion [MP] = 6.1%) and through chronic illness burden (β = 0.003; 95% CI, 0.000-0.006; *P* = .04; MP = 1.5%). Chronic medical illness burden (β = 0.025; 95% CI, 0.001-0.050; *P* = .04; MP = 12.6%) and presence of a cognitive disorder (β = 0.027; 95% CI, 0.003-0.051; *P* = .03; MP = 13.6%) mediated the association.

**Conclusions and Relevance:**

The risk factors shared by older adult couples may mediate approximately one-third of the spousal risk of depressive disorders. Identification of and intervention in the shared risk factors of depression among older adult couples may reduce the risk of depressive disorders in the spouses of older adults with depression.

## Key Points

**Question:**

Do the risk factors shared between older couples mediate their shared risk of depressive disorders?

**Findings:**

In this cohort study of 956 elderly couples, social-emotional support, chronic illness burden, and the presence of a cognitive disorder that were shared between couples mediated almost one-third of the spousal risk of depressive disorder.

**Meaning:**

These findings suggest that identification and intervention of the shared risk factors of depressive disorders within older couples may reduce the risk of depressive disorders in the spouses of people with depression.

## Teaser

This cohort study of elderly couples examines shared risk factors for depressive disorders and whether these factors mediate the shared risk of depressive disorders.

## Introduction

Depression is a significant public health problem for older adults and is associated with a substantial individual and societal burden.^1^ Depression in older adults is associated with decreased functional status, low perceived quality of life, increased use of medical services, decreased adherence to treatment plans, and higher admission rates to long-term care facilities.^1,2^ Thus, identifying individuals at high risk for geriatric depression and implementing preventive strategies may be important in this aspect of the public health burden of late-life depression.^1^

Previous studies^3,4,5,6,7^ have shown that having a partner with depression may increase an individual’s risk of depression^3,4,5,6^ (1.58-fold^3^ to 2.08-fold^7^). This spousal concordance of depression has been explained by assortative mating, cohabitation effects,^6,8^ and emotional contagion.^9^ Assortative mating refers to the tendency of mate selection based on the similarity of individual characteristics, including values and personality traits.^6^ Cohabitation effects explicate the significance of common household environmental influences, including the sharing of numerous lifestyle aspects after marriage.^6,9,10,11,12^ Emotional contagion shows that the mood of one person can lead to similar affect in another.^6,9^ In older adult couples, cohabitation effects may be more influential according to the duration of marriage, which may explain the difference in mechanism between older adult couple depression and that of young couples. Social interaction,^13,14^ physical health,^14,15,16^ or marital quality^13^ are known to affect depressive symptoms among older adult couples. These factors shared within couples may act as a mediator that links depression in couples. However, to our knowledge, no study has directly investigated the mediation of shared risk factors in couples regarding the risk of depressive disorders associated with spousal depressive disorders in the older adult population. Our objective was to identify the shared risk factors in couples and examine their mediating roles in the shared risk of depressive disorders among elderly couples in a population-based couple cohort study.

## Methods

### Study Design and Participants

We acquired data from the Korean Longitudinal Study on Cognitive Aging and Dementia (KLOSCAD).^17^ The KLOSCAD is an ongoing, nationwide, multicenter prospective cohort study of 6818 community-dwelling Koreans who were randomly sampled from the residents of 13 districts across South Korea. The inclusion criterion was age of 60 years or older, and there were no exclusion criteria. The baseline assessment was conducted from November 2010 to October 2012 with biannual follow-ups. At the fourth follow-up assessment (from January 1, 2019, to February 28, 2021), we constructed a spousal cohort (KLOSCAD-S) that consisted of the spouses of the KLOSCAD participants. The spouses of the 863 KLOSCAD participants responded. In the case of the 121 couples included in the original KLOSCAD cohort, 93 couples also completed their fourth follow-up assessment. We assigned these 93 participants who were enrolled later as spouses, resulting in a total of 956 participants in KLOSCAD-S.^18^ We introduced the KLOSCAD-S in a previous study,^18^ which overlaps in study design, covariates, and statistical analysis with this study. The study protocol was explained to all participants, each of whom provided written informed consent. The study protocol was approved by the institutional review board of the Seoul National University Bundang Hospital. This report followed the Strengthening the Reporting of Observational Studies in Epidemiology (STROBE) reporting guideline for observational studies.

### Assessment of Covariates

Research nurses evaluated the participants’ demographic characteristics (age, sex, and years of formal education). A history of heavy alcohol use was defined as the average lifetime amount of alcohol use over 21 standard units per week. Physical inactivity was defined as less than 2.5 hours of moderate activity per week and less than 1.25 hours of vigorous activity per week according to the World Health Organization’s recommendations on the minimum amount of activity that confers health benefits.^19^ We evaluated the burden of comorbid chronic medical illnesses, including hearing loss, smoking, hypertension, diabetes, dyslipidemia, cerebrovascular accident, history of head injury, and obesity using the Cumulative Illness Rating Scale (CIRS),^20^ which combines the morbidity of chronic medical problems of 14 organ systems into a single comprehensive score. The CIRS has been reported to be a valid indicator of health status in geriatric patients.^21^

We evaluated social-emotional and tangible support using the Medical Outcomes Study Social Support Survey (MOS-SSS), which is a brief, self-administered, multidimensional social support survey developed from the Medical Outcomes Study.^22^ On the basis of the 2-factor model,^23,24^ we defined social-emotional support using the items of emotional, informational, affectionate support, and positive social interaction. We defined tangible support using items of tangible support of the MOS-SSS. Each support has a score ranging from 0 to 100, with higher scores indicating better social support.^25^

Geriatric psychiatrists conducted face-to-face standardized diagnostic interviews and performed physical and neurologic examinations of every participant using the Korean version of the Consortium to Establish a Registry for Alzheimer Disease (CERAD-K) Assessment Packet Clinical Assessment Battery.^26^ Trained research neuropsychologists administered neuropsychological tests by means of the CERAD-K Neuropsychological Assessment Battery, which includes the Mini-Mental State Examination.^27^ We defined cognitive disorder as having mild cognitive impairment or dementia. Through diagnostic consensus conferences, a panel of geriatric psychiatrists diagnosed dementia and mild cognitive impairment according to the *Diagnostic and Statistical Manual of Mental Disorders* (Fourth Edition) (*DSM-IV*) criteria^28^ and the consensus criteria proposed by the International Working Group,^29^ respectively.

### Assessment of Depression

Geriatric psychiatrists assessed all participants through face-to-face standardized diagnostic interviews using the Korean version of the Mini International Neuropsychiatric Interview (MINI-K).^10^ A panel of geriatric psychiatrists diagnosed major depressive disorder (MDD) and minor depressive disorder (mDD) using the *DSM-IV* criteria.^28^ They also diagnosed subsyndromal depression (SSD) using the operational criteria.^30^ The operational diagnostic criteria for SSD were as follows: (1) the occurrence of 2 or more symptoms of depression listed in criterion A for a major depressive episode in the *DSM-IV* within the same 2-week period; (2) the presence of at least a depressed mood or anhedonia; (3) each depressive symptom should be present for more than a half day or more than 7 days during the 2-week period; (4) participants must not fulfill the criteria for the diagnosis of MDD or mDD; (5) the symptoms must not be due to the direct physiologic effects of a substance or a general medical condition; (6) the symptoms must not be attributable to bereavement, dementia, or schizophrenia and other psychotic disorders; and (7) there should not be a history of the occurrence of a manic or hypomanic episode. We defined depressive disorder as having MDD, mDD, or SSD. History of mood disorders, MDD, mDD, SSD, or bipolar disorder was evaluated by participants’ self-report and by previous MINI-K diagnoses during the follow-up period (in the case of KLOSCAD participants). We also evaluated the severity of depressive symptoms using the revised Korean version of the Geriatric Depression Scale (GDS).^31^

### Statistical Analysis

In a cross-sectional analysis, we compared the demographic and clinical characteristics between couples in whom the KLOSCAD participants (index participants) had depressive disorders and couples in whom the KLOSCAD participants did not have a depressive disorder, using Pearson χ^2^ tests for categorical variables and 2-tailed, unpaired *t* tests for continuous variables. We examined the agreement of the demographic and clinical characteristics in couples using the intraclass correlation coefficient for continuous variables and the κ coefficient for categorical variables.

We used binary logistic regression analyses to examine the association between index participants’ depressive disorders and the risk of depressive disorders among their spouses. We added spouses’ clinical characteristics with significant concordance in couples to the logistic regression model as independent variables. Next, we examined an additional binary logistic regression model to investigate the differential association of index participants’ depressive disorders with and without past mood history.

We then evaluated the mediating role of the significant factors shared in couples (from the logistic regression model) on the association between index participants’ depressive disorder and the risk of their spouses’ depressive disorders and depressive symptoms using structural equation modeling as follows:

Y = c + εX + β_1_M_1_ + β_2_M_2_ + β_3_M_3_ + … + β_k_M_k_

M_k_ = d + α_k_X for k = 1, …, K,

where X, M, and Y are the exposure, mediators, and outcomes, respectively. We included mediator-mediator interactions in the model if the mediators affect one another; for 2 mediators with interactions, the model becomes as follows:

M_i_ = d + α_i_X

M_j_ = d + α_j_X + γijMi.

We then estimated the indirect and direct effects of exposure X on outcome Y as follows:

Direct effect = ε

Indirect effect = α_1_β_1 _+ α_2_β_2 _+ α_3_β_3 _+ ∙∙∙ α_i_γijβ_j _+ α_k_β_k_.

Significant indirect effects were confirmed with 95% CIs using the adjusted bootstrap percentile method. All statistical analyses were performed using the lavaan package in R software, version 4.2.2 (R Foundation for Statistical Computing). *P* values were 2-sided, with *P* < .05 considered statistically significant.

## Results

The demographic and clinical characteristics of the index and spousal participants are given in Table 1. All couples were heterosexual. Compared with the index participants, the spouse participants were younger (mean [SD] age, 75.1 [5.0] years; age range, 67-94 years for index participants; mean [SD] age, 73.9 [6.1] years; age range, 55-94 years for spouses; *P* < .001) but equally educated (mean [SD], 10.2 [5.0] years of education for index participants; mean [SD], 9.9 [5.0] years of education for spouses; *P* = .16). Among the 956 index participants, 82 (8.6%) had depressive disorders. As summarized in Table 2, age, educational level, social-emotional support, and Mini-Mental State Examination scores were highly concordant within couples regardless of whether the index participants had depressive disorders. However, a history of heavy alcohol use, physical inactivity, CIRS score, tangible support, cognitive disorder, and GDS score were concordant in couples only when the index participants did not have depressive disorders.

**Table 1.** Demographic and Clinical Characteristics of the Participants^a^

Characteristic	Index participants			Spouse participants		
	IDD+ (n = 82)	IDD− (n = 874)	*P* value^b^	SDD+ (n = 82)	SDD− (n = 874)	*P* value^b^
Age, mean (SD), y	76.3 (5.3)	75.0 (5.0)	.02	75.4 (6.3)	73.8 (6.1)	.02
Educational level, mean (SD), y	9.9 (5.0)	10.3 (5.0)	.50	9.9 (5.2)	9.9 (5.0)	.94
Sex						
Female	40 (48.8)	345 (39.5)	.13	42 (51.2)	529 (60.5)	.13
Male	42 (51.2)	529 (60.5)		40 (48.8)	345 (39.5)	
Lifetime amount of alcohol use, mean (SD), SU/wk	2.1 (8.4)	2.5 (7.3)	.65	3.3 (9.7)	2.5 (9.1)	.43
Current alcohol use	20 (24.4)	272 (31.1)	.25	27 (32.9)	219 (25.1)	.15
History of heavy alcohol use^c^	8 (9.8)	109 (12.5)	.59	6 (7.3)	76 (8.7)	.83
MET, mean (SD), h/wk	14.9 (22.0)	23.8 (34.5)	.001	19.9 (24.6)	21.2 (29.7)	.65
Physical inactivity^d^	47 (57.3)	278 (31.8)	<.001	34 (41.5)	314 (35.9)	.38
Social-emotional support score, mean (SD)^e^	59.6 (27.7)	77.6 (21.2)	<.001	70.6 (23.7)	77.6 (21.8)	.007
Tangible support score, mean (SD)^f^	73.5 (24.9)	84.0 (19.7)	<.001	75.0 (25.0)	82.3 (20.8)	.01
CIRS score, mean (SD)	9.4 (3.6)	6.4 (3.2)	<.001	6.7 (2.8)	5.7 (3.2)	.01
MMSE score, mean (SD)	25.3 (4.3)	27.0 (2.9)	<.001	25.7 (4.4)	26.4 (3.3)	.18
Cognitive disorders^g^	35 (42.7)	129 (14.8)	<.001	34 (41.5)	194 (22.2)	<.001
GDS score, mean (SD)^h^	18.4 (6.1)	7.2 (5.3)	<.001	11.7 (6.8)	8.1 (6.1)	<.001
History of mood disorders^i^	66 (80.5)	146 (16.7)	<.001	17 (20.7)	72 (8.2)	<.001
Current depressive disorders						
Major depressive disorder	7 (8.5)	0 (0.0)	<.001	1 (1.2)	5 (0.6)	<.001
Minor depressive disorder	12 (14.6)	0 (0.0)		2 (2.4)	7 (0.8)	
Subsyndromal depression	63 (76.8)	0 (0.0)		19 (23.2)	57 (6.5)	

Abbreviations: CIRS, Cumulative Illness Rating Scale; GDS, Geriatric Depression Scale; IDD+, index participants with depressive disorders; IDD−, index participants without depressive disorders; MET, metabolic equivalent task; MMSE, Mini-Mental State Examination; SDD+, spouses of the IDD+ participants; SDD−, spouses of the IDD− participants; SU, standard unit.

^a^ Data are presented as number (percentage) of study participants unless otherwise indicated.

^b^ The 2-tailed, unpaired *t* test was used for continuous variables and the χ^2^ test for categorical variables.

^c^ Mean lifetime amount of alcohol use of 21 SU/wk or more.

^d^ Less than 2.5 hours/wk of moderate activity and less than 1.25 hours/wk of vigorous activity.

^e^ Sum of the emotional support, informational support, positive social interaction, and affectionate support scores of the Medical Outcomes Study Social Support Survey.

^f^ Tangible support from the Medical Outcomes Study Social Support Survey.

^g^ Mild cognitive impairment or dementia.

^h^ Missing values: 2 from the IDD+ group, 1 from the IDD− group, 2 from the SDD+ group, and 3 from the SDD− group.

^i^ Major depressive disorder, minor depressive disorder, subsyndromal depression, or bipolar disorder.

**Table 2.** Concordance of Demographic and Clinical Characteristics Within Couples

Characteristic	Between IDD+ and SDD+			Between IDD− and SDD−		
	Difference^a^	Concordance^b^	*P* value	Difference^a^	Concordance^b^	*P* value
Age, y	0.94 (5.37)	0.571	<.001	1.17 (4.63)	0.636	<.001
Educational level, y	0.00 (5.10)	0.505	<.001	0.35 (4.70)	0.561	<.001
Lifetime amount of alcohol use, SU/wk	−1.15 (13.29)	−0.078	.76	0.07 (12.00)	−0.060	.96
Current alcohol use	−7.0 (−8.5)	−0.035	.75	53.0 (6.0)	−0.018	.60
History of heavy alcohol use^c^	2.0 (2.5)	−0.091	.40	33.0 (3.8)	−0.078	.02
MET, h/wk	−4.97 (30.85)	0.124	.13	2.62 (39.80)	0.236	<.001
Physical inactivity^d^	13.0 (15.8)	0.120	.26	−36.0 (−4.1)	0.220	<.001
Social-emotional support score^e^	−9.92 (31.54)	0.212	.02	−0.05 (25.20)	0.312	<.001
Tangible support score^f^	−1.51 (33.00)	0.126	.14	1.64 (25.83)	0.190	<.001
CIRS score	2.73 (4.52)	−0.001	.51	0.69 (4.17)	0.157	<.001
MMSE score	−0.40 (4.85)	0.386	<.001	0.68 (3.78)	0.241	<.001
Cognitive disorders^g^	1.0 (1.2)	−0.126	.26	−65.0 (−7.4)	0.153	<.001
GDS score	6.84 (8.37)	0.104	.12	−0.87 (7.23)	0.201	<.001

Abbreviations: CIRS, Cumulative Illness Rating Scale; GDS, Geriatric Depression Scale; IDD+, index participants with depressive disorders; IDD−, index participants without depressive disorders; MET, metabolic equivalent task; MMSE, Mini-Mental State Examination; SDD+, spouses of the IDD+ participants; SDD−, spouses of the IDD− participants; SU, standard unit.

^a^ Data are presented as mean (SD) for continuous variables and number (percentage) for categorical variables.

^b^ Intraclass correlation coefficient for continuous variables and κ coefficients for categorical variables.

^c^ Mean lifetime amount of alcohol use of 21 SU/wk or more.

^d^ Less than 2.5 hours/wk of moderate and less than 1.25 hours/wk of vigorous activity.

^e^ Sum of the emotional support, informational support, positive social interaction, and affectionate support scores of the Medical Outcomes Study Social Support Survey.

^f^ Tangible support from Medical Outcomes Study Social Support Survey.

^g^ Mild cognitive impairment or dementia.

The spouses of index participants with depressive disorder were older, were more likely to have a higher medical illness burden and cognitive disorder, and had less social-emotional and tangible support than the spouses of participants without depressive disorder. The spouses of participants with depressive disorder exhibited higher symptoms of depression and were more likely to have depressive disorders than the spouses of participants without depressive disorder (22 [26.8%] vs 69 [7.9%], *P* < .001) (Table 1), indicating that having a depressive disorder may be associated with an increased risk of depressive disorder in one’s spouse.

In the logistic regression models, the spouses of participants with depressive disorder had an approximately 4 times higher risk of depressive disorders than the spouses of participants without depressive disorder (model 2; odds ratio [OR], 3.89; 95% CI, 2.06-7.19; *P* < .001) (Table 3). This association remained significant when the factors that were concordant in couples were adjusted. Among the factors that were concordant in couples, social-emotional support score, cognitive disorder, and CIRS score were independently associated with the risk of depressive disorders in spousal participants (model 2) (Table 3).

**Table 3.** Association of Depressive Disorders of Index Participants on the Risk of Depressive Disorders of Their Spouses

Factor	Model 1^a^		Model 2^a^	
	OR (95% CI)	*P* value	OR (95% CI)	*P* value
Depressive disorder of index participants^b^	4.60 (2.59-7.98)	<.001	3.89 (2.06-7.19)	<.001
Shared factors				
Heavy alcohol use^c^	NA	NA	1.86 (0.70-4.66)	.20
Physical inactivity^d^	NA	NA	1.19 (0.71-1.99)	.50
Social-emotional support^e^	NA	NA	0.97 (0.96-0.99)	<.001
Tangible support^f^	NA	NA	2.40 (0.56-10.59)	.24
Cognitive disorders^g^	NA	NA	2.73 (1.65-4.51)	<.001
CIRS, total	NA	NA	1.26 (1.17-1.37)	<.001

Abbreviations: CIRS, Cumulative Illness Rating Scale; NA, not applicable; OR, odds ratio; SU, standard unit.

^a^ Binary logistic regression analyses adjusted for age, sex, and educational level.

^b^ Major depressive disorder, minor depressive disorder, or subsyndromal depression.

^c^ Mean lifetime amount of alcohol use of 21 SU/wk or more.

^d^ Less than 2.5 hours/wk of moderate activity and less than 1.25 hours/wk of vigorous activity.

^e^ Sum of the emotional support, informational support, positive social interaction, and affectionate support scores of the Medical Outcomes Study Social Support Survey.

^f^ Tangible support from the Medical Outcomes Study Social Support Survey.

^g^ Mild cognitive impairment or dementia.

Among the 82 index participants with depressive disorders, 16 (19.5%) were newly diagnosed with depressive disorders without a history of depressive disorders, and 66 (80.5%) had a history of depressive disorders. Both index participants with depressive disorders with and without a history of depressive disorders were associated with the risk of depressive disorders in their spouses (without a history: OR, 6.40; 95% CI, 2.10-18.10; *P* < .001; with a history: OR, 4.20; 95% CI, 2.20-7.70; *P* < .001), and these ORs were not significantly different from each other. Thus, we did not consider the influence of the mood history of index participants on spouses’ depression in subsequent analyses.

In the structural equation model with multiple mediators (Figure, A), social-emotional support mediated the association between depressive disorder in the index participants and their spouses’ risk of depressive disorder by itself (standardized coefficient β = 0.012; 95% CI, 0.001-0.024; *P* = .04; mediation proportion [MP] = 6.1%) and by lowering the CIRS score (β = 0.003; 95% CI, 0.000-0.006; *P* = .04; MP = 1.5%). Spousal participants’ cognitive disorder and CIRS score also mediated the association between depressive disorder in the index participants and their spouses’ risk of depressive disorders (cognitive disorders: β = 0.027; 95% CI, 0.003-0.051; *P* = .03; MP = 13.6%; CIRS score: β = 0.025; 95% CI, 0.001-0.050; *P* = .04; MP = 12.6%). The direct and total effects of index participants’ depressive disorders on the risk of spouses’ depressive disorders were also significant (β = 0.122; 95% CI, 0.036-0.209; *P* = .005 for direct effect [61.6% of total effect]; β = 0.198; 95% CI, 0.116-0.280; *P* < .001 for total effect). In the association between depressive disorders of the index participants and their spouses’ GDS score, social-emotional support and cognitive disorder mediated the association. The CIRS score mediated the association by being influenced by social-emotional support (Figure, B).

**Figure.**
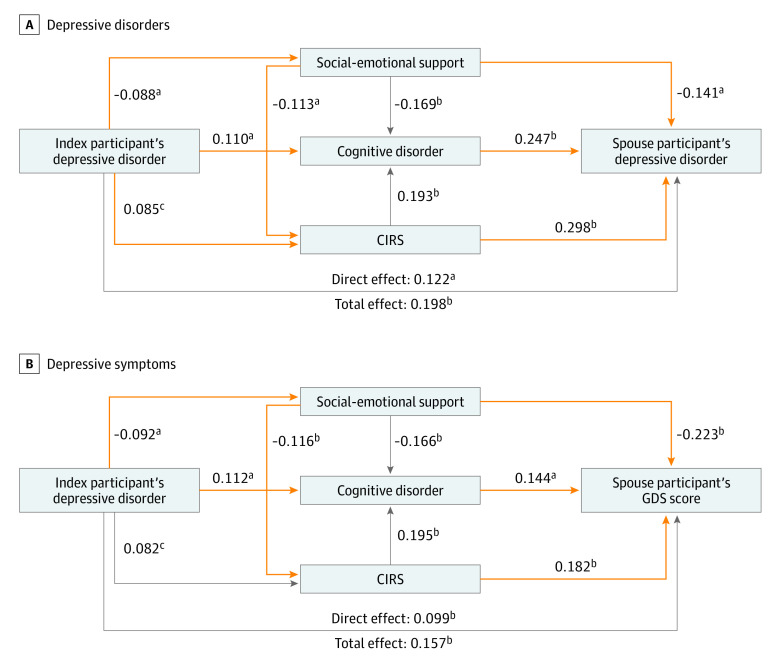
Mediating Role of the Factors Shared Within Couples in the Shared Risk of Depressive Disorders Direct and indirect associations between index participants’ depressive disorders and the risk of depressive disorders (A) and depressive symptoms (B) of spouses are shown along with standardized coefficients. All lines indicate statistically significant associations. The orange lines indicate significant mediation pathways with 95% CIs using the adjusted bootstrap percentile method. The Cumulative Illness Rating Scale (CIRS) scores were square-root transformed for normal distribution. GDS indicates Geriatric Depression Scale. ^a^*P* < .01. ^b^*P* < .001. ^c^*P* < .05.

## Discussion

This study found that index participants’ depressive disorders were associated with spouses’ depressive disorders and that this association was mediated by social-emotional support, a cognitive disorder, and cumulative chronic illness burden, which were shared in couples. In addition, having a history of mood episodes did not differentially affect the risk of depressive disorders of spouses compared with incident depression.

Our results showed a relatively higher OR for depression associated with spousal depression (OR, 3.89) than previous studies^6,7^ that did not limit the study population to old age (ORs, 2.08 for depression^7^ and 2.91 for mental disorders^6^). It is possible that not only old age itself but also the unique mechanism of depression in older adult couples may have played a role in contributing to an increased risk of mental disorders.

In the older adult population, reduced social networks due to physical, economic, and social factors are associated with limited social support.^14^ As a person ages, social networks and connectedness may be limited to family members,^14^ particularly the spouse, who serves as the main provider of social-emotional support. When a person has a depressive disorder, the social-emotional support he or she provides to his or her spouse may be reduced, which may influence the occurrence of depressive disorder in the spouse. Moreover, if the spouse of a person with a depressive disorder is the caregiver,^6,20^ the spouse’s social network external to his or her family may not be as accessible. Interestingly, tangible support was not associated with spousal depressive disorder when considered with social-emotional support in the model (Table 3). This finding may suggest that the pivotal targets for depression prevention in older adult couples may be formal social-emotional support outside the family and the coping strategies of an informal caregiver,^6,32,33^ which may be more efficacious when added to tangible support (material aid or behavioral assistance), such as long-term care services.

Social-emotional support mediated the association of couple depression by itself and by increasing the CIRS score. The CIRS score also independently mediated the association of couple depression. In older adult couples, shared environmental factors, diet,^34^ lifestyle,^35^ and health-related behaviors may influence the concurrence of chronic medical conditions in couples.^36,37^ Examples include coronary heart disease,^38,39^ hypertension,^38^ hyperlipidemia,^7^ lung cancer,^40^ diabetes,^41^ musculoskeletal health,^42^ and mental health.^36,43,44^ These chronic medical conditions are also known to be associated with the risk of late-life depression.^6,45,46^ Furthermore, one partner’s depressive disorder may affect the other partner’s chronic medical condition as one of the stressors (to the spouse) or by the loss of proper management, which may influence the occurrence of depressive disorder in the spouse. Therefore, lowering the burden of chronic medical illness in couples and proper management of chronic medical illness may be important to reduce the co-occurrence of depression in older adult couples.

Cognitive disorders mediated couple depression in the largest proportion among significant mediating variables. Recently, the Rotterdam study^47^ reported that higher cognitive reserve, defined as the common variance across cognitive tests, may be a protective factor for late-life depression, which is in line with several previous studies^48,49,50^ that suggested that low cognitive reserve may increase the risk of late-life depression. Cognitive and brain reserve coping mechanisms are particularly important in regions that are related to neuromodulation of the serotonin system.^48^ In our study, the mediating role of cognitive disorder between couple depression may reflect the impaired coping or adaptation ability of the person against spousal depression,^47^ which may also relate to one’s own depression. Of course, we should consider the cross-sectional design of this study, which may limit the interpretation of the directionality between cognitive disorders and depression.

These 3 shared risk factors mediated approximately one-third of the spousal risk of depressive disorders. Marital environments, including household income, marital quality,^13^ negative life events, and actual caregiving status,^51^ and individual factors, such as perceived stress, coping strategies,^52^ other psychiatric morbidity,^3^ personality,^3^ medication use, and shared genetic disposition,^4^ may serve as other potential mediators.

To our knowledge, this is the first study to reveal how the association among depressive disorders and shared risk factors is structured within community-dwelling older adult couples based on psychiatrist diagnosis. We diagnosed depressive disorders with psychiatrists’ diagnostic interviews based on the MINI-K, which may confirm the diagnostic validity. Previous studies that investigated couple depression in large population samples usually adopted a survey,^5,16,53^ scale,^4,9,13,14,15,16,54^ or medical records^3,6,36^ as diagnostic confirmation. In contrast with previous studies^3,5,53^ focusing on syndromal depression (MDD or mDD), we included SSD as a depressive disorder. Subsyndromal depression has a 2.4-fold higher prevalence than that of syndromal depression, which is also associated with poor outcomes in late life.^30^ Therefore, the current study may better reflect real-world depression in the older adult population.

### Limitations

This study has several limitations. First, it used a cross-sectional design, which may not confirm causality and temporal order. However, the association of chronic or recurrent depression among the index participants with spousal depressive disorder was not significantly different from the association of first-onset depression among the index participants with spousal depression, which was why we focused on the cross-sectional association. Second, marital discord could be a contributor to depression in couples,^36^ but we had no information on the quality of the relationship within the partnership. Some potentially mediating factors also were not considered, as discussed earlier. Third, we could not perform separate analyses for syndromal depression because of the small number of individuals with this condition. Fourth, we did not consider acute medical conditions or fluctuating health status that may contribute to current depression but are not reflected in the CIRS.

## Conclusions

This cohort study may imply that the depression status of a spouse should also be treated as an indication of the risk of depression in the older adult population.^5,6^ Establishing an external social-emotional support system, active prevention of cognitive disorders, and proper management of chronic medical illness for elderly couples should be considered in the policy making process to reduce the burden of geriatric depression.^6^ Because of limited social networks, multiple chronic medical comorbidities, and high prevalence of cognitive impairment in the older adult population, active intervention to address these mediating factors may be more important than in the younger population.

## Notes

Supplement.
Data Sharing Statement
