# Supplementary material for: Shared Risk Factors for Depressive Disorder Among Older Adult Couples in Korea
Source: JAMA Netw Open. 2023 Apr 14;6(4):e238263. doi: 10.1001/jamanetworkopen.2023.8263 (PMC10105310; doi:10.1001/jamanetworkopen.2023.8263)
Supplement: Supplement. — Data Sharing Statement [file jamanetwopen-e238263-s001.pdf]

Data Sharing Statement

Han. Shared Risk Factors for Depressive Disorder Among Older Adult Couples in Korea. *JAMA Netw Open*. Published April 14, 2023. doi:10.1001/jamanetworkopen.2023.8263

Data
Data available: No

Additional Information
Explanation for why data not available: The data that support the findings of this study are available from the corresponding author, Dr K. W. Kim, upon reasonable request.
